# Pharmacology and therapeutics resource session attendance and academic performance of pre-clerkship medical students in problem-based learning curricula

**DOI:** 10.1186/s12909-019-1699-3

**Published:** 2019-07-18

**Authors:** Khalid Ahmed Jassim Al Khaja, Yasin Tayem, Henry James, Ahmed Jaradat, Reginald Paul Sequeira

**Affiliations:** 1 Department of Pharmacology & Therapeutics, Arabian Gulf University, Manama, P.O. Box 22979, Kingdom of Bahrain; 2 Department of Family & Community Medicine, College of Medicine & Medical Sciences, Arabian Gulf University, Manama, Kingdom of Bahrain

**Keywords:** Pre-clerkship phase, Class attendance, Pharmacology achievement, Medical students, PBL curriculum, Kingdom of Bahrain

## Abstract

**Background:**

The relationship between large-group classroom attendance by students and test achievement in problem-based learning (PBL) curricula is unclear. This study examined the correlation between attendance at resource sessions (hybrid lectures in the PBL curriculum) and test scores achieved in pharmacology and determined whether the score achieved was related to student gender.

**Methods:**

A cross-sectional observational study over one academic year of 1404 pre-clerkship medical students was performed. Class attendance during pharmacology resource sessions and MCQ test scores achieved in pharmacology were analysed.

**Results:**

The percentage of students’ attendance in resource sessions declined over three years of the programme, from 78.7 ± 27.5 in unit I to 22.1 ± 35.6 (mean ± SD) in unit IX. A significant but weakly positive correlation was evident between attendance and achievement in pharmacology (*r* = 0.280; *p* < 0.0001). The mean score of the students who attended > 50% of the resource sessions was significantly higher (*p* < 0.0001). Students who attended ≤50% were more likely to achieve lower *tertile* scores. The mean score achieved and the number of higher *tertile* scorers were higher among students who attended > 50% of the resource sessions. Although female students’ attendance was significantly higher, no significant gender-related differences in either mean scores or top grades achieved were found.

**Conclusions:**

In a PBL curriculum, the classroom attendance of students in pharmacology declined during the pre-clerkship phase. A weak positive correlation was found between attendance and academic achievement, as measured by MCQ test scores. Factors other than motivation and attendance may confound gender-based academic performance and merit further research.

## Background

It is well known that students’ absenteeism in class is a universal phenomenon that appears to transcend beyond the country, university, and subject discipline [1–3]. It is considered a challenge in curriculum implementation in tertiary education worldwide [2, 3]. Absenteeism indicates poor motivation for learning [2], affects student retention in programmes [4, 5], and has an adverse impact on students’ academic performance [6–12]. Student absenteeism has been attributed to faculty, student, and learning-environment-related factors [13–15]. Previous studies have confirmed that attendance and performance are related even after adjustments are made for several student-related variables [16–19].

There is a consensus among published studies that the absenteeism of medical students during the preclinical (pre-clerkship) phase [6–8] and the clinical (clerkship) phase [9–12, 20] results in poor academic and clinical achievements. In medical schools implementing traditional curricula, attending lectures appears to be crucial for achieving pharmacology-learning outcomes [7, 8]. However, much less information is available for integrated medical curricula, particularly from schools in which an integrated student assessment strategy is practised. The effect of class attendance on examination scores for male and female medical students is debatable [21].

Since its inception in the early 1980s, the College of Medicine and Medical Sciences at Arabian Gulf University (CMMS-AGU) has adopted a problem-based learning (PBL) curriculum that is divided into three phases: phase I (premedical, 1 year), phase II (pre-clerkship, 3 years), and phase III (clerkship, 2 years) [22]. Recently, we reported a significant positive correlation between student attendance in structured classroom educational activities and the total scores achieved by students on the objective structured practical examination (OSPE), which assessed prescribing skills [22]. However, to our knowledge, the relationship between students’ attendance in resource sessions (hybrid lectures in PBL) and student performance on written tests (comprising multiple-choice questions and short-answer questions to assess knowledge) has not been evaluated in any preclinical learning environments of medical schools that implement a PBL curriculum.

This study was conducted to (a) determine the trend of students’ classroom absenteeism during the three years of the pre-clerkship phase, (b) measure the correlation between students’ attendance at resource sessions and their performance in pharmacology and therapeutics in the pre-clerkship learning environment, and (c) determine whether such attendance-related test performance is affected by gender.

## Methods

### Setting

The study was conducted at CMMS-AGU among pre-clerkship medical students over one academic year (September 2013 to June 2014). The pre-clerkship phase (unit phase) comprises 94 weeks; each week, clinical problems are presented to students [22]. Of these problems, 64 had scheduled resource sessions in pharmacology and therapeutics, given in the form of large-group presentations by faculty (Table 1).

**Table 1:** Number of pharmacology resource sessions and time allocated per unit for medical students at the pre-clerkship phase

Pre-clerkship Phase					
*Theoretical*		Number of students	Number of problems/unit	Number of pharmacology resources/unit	Time allocated for pharmacology resources/unit (hours:minutes)
Year	Unit				
2	I	182	11	9	5:40
2	II	182	8	4	2:15
2	III	182	13	9	4:50
3	IV	143	12	9	4:20
3	V	143	12	9	3:55
3	VI	143	10	7	3:25
4	VII	152	9	4	1:55
4	VIII	152	12	9	4:45
4	IX	152	6	4	2:50
			93	64	31:55
*Laboratory skill*					16:00
*Dry laboratory*					4:00
*Total hours*					51:55
*Credit hours per semester*					1:35

### Pre-clerkship teacher-centred activities

At CMMS, pre-clerkship educational activities include tutorials, hybrid lectures (PBL resource sessions), laboratory skills and demonstrations, professional clinical skills, and community health activities training. A typical schedule of weekly educational activities is shown in Table 2. Attendance at these structured educational activities was mandatory for students, except for the resource sessions. Each PBL resource session lasts for an hour, with intervals of at least two days between sessions to allow the students to spend time meeting their problem-related learning needs. The resource session was typically shared by two faculty from basic or clinical science disciplines. During these sessions, the faculty deliver interactive lectures to a large group of students, with a primary focus on discipline content related to the problem of each week [23].

**Table 2:** Schedule of weekly structured educational activities for pre-clerkship medical students^*^

Theoretical educational activity	Students’ attendance	Time	Sunday	Tuesday	Thursday	Sunday
Tutorial^a^	Compulsory	Forenoon	2–3 h	–	2–3 h	2–3 h
Resource session^b^	Optional	Noon	–	1 h^c^	1 h^c^	1 h^d^

^a^Small group activity of 9–11 students

^b^Large group activity in lecture theatres

^c^Time shared by 1–3 faculty resource persons from different disciplines; d, review session with attendance of all students and discipline’s resource person

^*^Other scheduled activities include laboratory skills, professional clinical skills, and community health activities

### Pre-clerkship student assessment

At the end of each unit in phase II of the MD programme, student assessment was composed of a comprehensive written test with at least 75 A-type multiple-choice questions (MCQs) and 4–5 integrated short-answer questions (SAQs), each with 6–8 subcomponents. An OSPE test comprising 30–35 stations was administered in all units except in unit IX. All end-unit tests included the following: approximately 10–16 MCQs, 2–3 SAQs integrated with basic and clinical disciplines, and 2–3 therapeutics-related OSPE stations (usually 1 prescription, 1 chart order, and 1 calculation or data interpretation station). The majority of test items included a vignette (clinical scenario or interpretation of graph or figure) and placed less emphasis on factual recall and more emphasis on the interpretation and application of knowledge. Some of the MCQ items were cluster-type items with a focus on interdisciplinary integration to ensure that the assessment was congruent with the integrated curriculum. Most of the MCQs and OSPEs were developed and evaluated by discipline experts (resource faculty for the course), whereas SAQs were generated by the unit committee structuring interdisciplinary integration.

The number of test items and the weight for pharmacology and therapeutics in each end-unit exam was proportional to the input into the curriculum and was identified in terms of learning objectives and outcomes. An examination blueprint approved by each unit committee was routinely used for planning the exam in terms of the weight of test items for each discipline. Generally, an estimated 10–15% weight was allocated for pharmacology and therapeutics in written components of the test.

The standard setting procedure for the written and OSPE exams is based on the modified Angoff method [24], determined individually by a panel of 6–8 judges who were the unit committee members responsible for the planning and implementation of units. A standardized score based on the mean “cut-off” score judged by the panellists was the basis on which the pass/fail decision was made. The passing score was 60% for all units.

The final grades, reported as percentage scores (transformed into letter grades), was based on end-unit written and OSPE scores, clinical professional skills exam scores, and continuous evaluation scores based on performance in small-group tutorials graded by a faculty facilitator. Each end-unit exam score was reported using the compensatory approach [25].

### Attendance and absenteeism monitoring

During the resource sessions, the students’ attendance was monitored based on their signatures on a paper-based attendance register.

### Performance monitoring

The performance of students in pharmacology and therapeutics for MCQ components of the end-unit test was assessed based on the optical mark recognition test form. The rate of absenteeism/attendance per unit and the MCQ scores in each unit were correlated.

### Operational definition

The resource session is used as an interchangeable phrase for large-group classroom sessions or classroom educational activities presented by content expert faculty members. Lower, mid, and higher *tertiles* represent students with ≤33.3, > 33.3% to ≤66.6 and > 66.6% scores (in pharmacology), respectively.

### Statistical analysis

Data were entered and analysed using SPSS Version 25 (IBM®-Bahrain). Variables are presented as counts and percentages or as means and standard deviations where applicable. Two independent samples t-tests were used to test the significant mean differences in student performance in pharmacology and therapeutic scores with regard to percentage of attendance and gender. The Pearson correlation coefficient was used to measure the linear relationship between the pharmacology score and percentage of attendance. A chi-square test was used to compare the proportions of students in each *tertile* category according to attendance and gender. Additionally, the chi-square test was used to measure the association between students’ performance in pharmacology, attendance, and gender. A *p*-value < 0.05 was considered statistically significant.

### Study approval

This study was approved by the Department of Pharmacology and Therapeutics Council for Course Evaluation.

## Results

A total of 1404 medical student data were evaluated in this study; 812 (57.4%) were females, and 592 (42.2%) were males.

### Resource session attendance

**Fig. 1:**
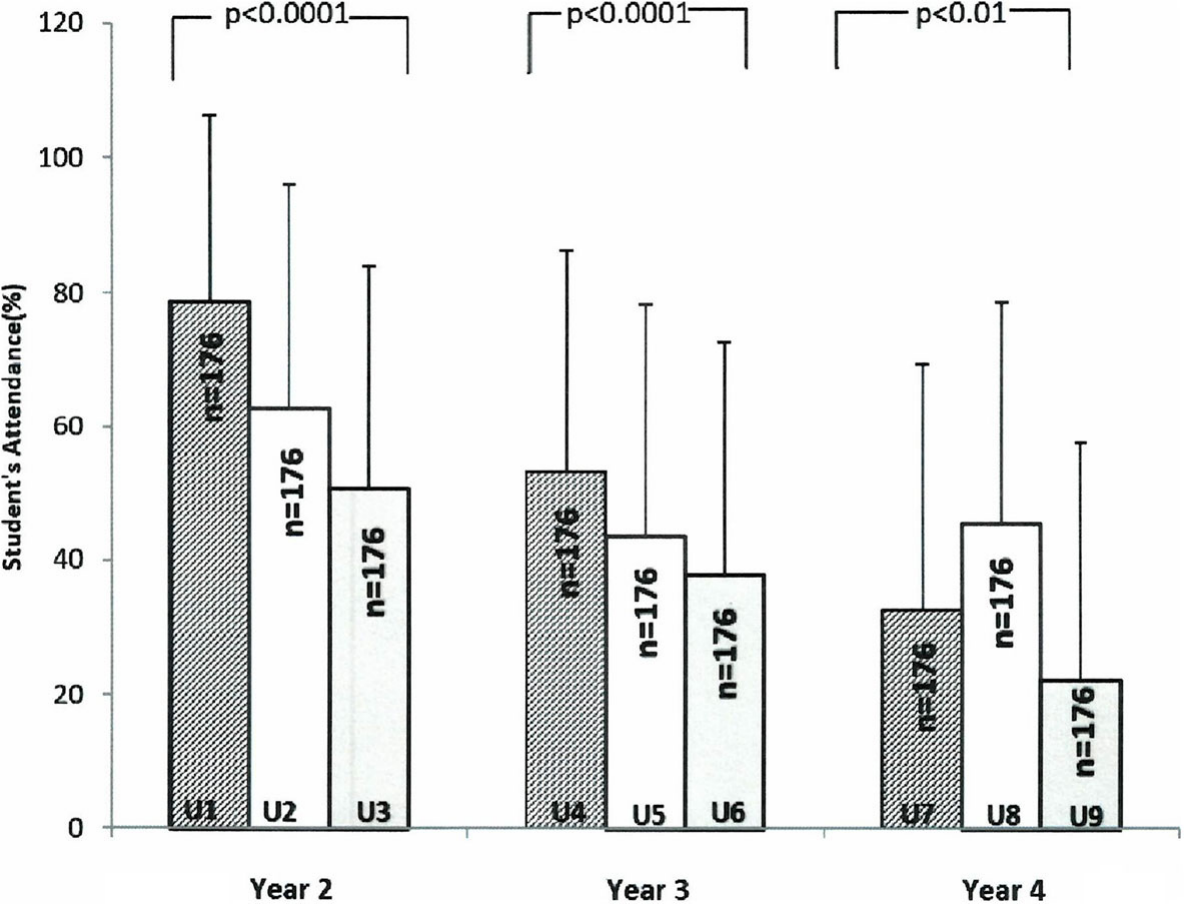
Resource session attendance (mean ± SD) pattern of pre-clerkship medical students in different units and years in a problem-based learning curriculum

Resource session attendance of medical students during the year of study is shown in Fig. 1. The mean percentage ± SD resource session attendance of second-year students in units I, II and III declined from 78.7% ± 27.5% in unit I to 50.8% ± 33.3% in unit III (*p* < 0.0001). Similarly, during unit IV to unit VI, the mean percentage of attendance of third-year students declined from 53.4% ± 33.0% (unit IV) to 37.8% ± 34.8% (unit VI; *p* < 0.0001). Additionally, the attendance of fourth-year students showed a decline from 32.6% ± 36.8% (unit VII) to 22.1 ± 35.6% (unit IX; *p* = 0.02), except for a transient increase in unit VIII.

### Test performance

The association between the percentages of attendance and academic achievement (MCQ score in pharmacology) is presented (Table 3). A significant but low positive correlation between the students’ resource session attendance and test achievement was evident by a correlation coefficient (r) value of 0.280 (*p* < 0.001). The association between attendance and performance for three cohorts of students representing years 2, 3, and 4 (pre-clerkship phase) was as follows: a) *r* = 0.240, *p* < 0.001 for 528 s-year students (units I, II, III); b) *r* = 0.267, *p* < 0.001 for 421 third-year students (units IV, V, VI); and c) *r* = 0.376, *p* < 0.001 for 455 fourth-year students (units VII, VIII, IX) (data not shown).

**Table 3:** The relationship between resource session attendance and performance in pharmacology MCQs score

Mean score of pharmacology performance per percentage of students’ attendance											
Year	Unit	No of students	0%	1–24%	25–49%	50–74%	75–99%	100%	Mean score ± SD	Correlation coefficient (г)	*p*-value
2	I	176	51.9 ± 14.1 (8)	46.1 ± 21.7 (6)	54.7 ± 14.1 (9)	50.3 ± 20.3 (28)	55.4 ± 17.3 (40)	62.9 ± 15.0 (85)	57.7 ± 17.3 (176)	0.264	< 0.001
2	II	177	63.1 ± 14.1 (21)	(0)	67.4 ± 13.8 (22)	64.4 ± 12.7 (33)	71.3 ± 12.7 (49)	73.9 ± 13.6 (52)	69.3 ± 13.8 (177)	0.268	< 0.001
2	III	175	42.6 ± 17.1 (19)	53.7 ± 18.5 (38)	56.8 ± 20.0 (19)	66.3 ± 15.8 (42)	66.7 ± 18.5 (41)	62.5 ± 15.0 (16)	59.7 ± 19.1 (175)	0.349	< 0.001
3	IV	141	40.9 ± 15.4 (22)	42.8 ± 18.0 (7)	43.9 ± 20.0 (33)	51.2 ± 22.0 (42)	52.9 ± 24.4 (17)	62.0 ± 23.3 (20)	49.2 ± 21.7 (141)	0.309	< 0.001
3	V	141	46.2 ± 16.6 (31)	47.3 ± 20.5 (28)	51.6 ± 21.3 (16)	53.8 ± 19.7 (22)	67.8 ± 19.3 (38)	70.8 ± 15.6 (6)	55.1 ± 21.0 (141)	0.421	< 0.001
3	VI	139	64.7 ± 17.3 (45)	66.7 ± 20.5 (18)	81.5 ± 18.7 (21)	85.6 ± 12.8 (30)	82.6 ± 17.2 (16)	92.6 ± 7.9 (9)	75.9 ± 19.2 (139)	0.493	< 0.001
4	VII	151	58.7 ± 18.6 (65)	(0)	58.3 ± 18.4 (35)	62.8 ± 20.9 (13)	75.5 ± 21.4 (16)	84.1 ± 10.9 (22)	64.4 ± 20.4 (151)	0.444	< 0.001
4	VIII	152	51.9 ± 23.9 (29)	60.4 ± 21.1 (16)	61.5 ± 21.3 (27)	60.2 ± 20.7 (31)	70.5 ± 19.9 (39)	75.3 ± 13.7 (10)	62.5 ± 21.8 (152)	0.309	< 0.001
4	IX	152	54.6 ± 17.1 (101)	(0)	60.0 ± 15.2 (20)	72.9 ± 19.1 (12)	(0)	70.7 ± 14.0 (19)	58.8 ± 17.8 (152)	0.360	< 0.001
		1404	54.6 ± 18.9 (341)	54.1 ± 20.7 (113)	59.1 ± 21.0 (202)	62.4 ± 21.0 (253)	67.2 ± 19.6 (256)	69.6 ± 16.9 (239)	61.5 ± 20.4 (1404)	0.280	< 0.001

Table 4 presents the lower, mid, and higher *tertile* percentages and the mean score achieved by students who attended ≤50% versus > 50% of the resource sessions. The mean score in pharmacology achieved by students who attended > 50% of the resource sessions was significantly higher than that of those with poor attendance (66.7% ± 19.6% vs. 56.4% ± 19.8%; *p* < 0.0001). The lower *tertile* score of students who attended ≤50 resource sessions was significantly higher than the lower *tertile* score of students who attended > 50 (14.6% vs. 6.6%; *p* < 0.0001), but there was no significant difference in the mean lower *tertile* score achieved. On the other hand, the higher mean *tertile* score and mean score achieved were substantially higher among students who attended > 50% of the resource sessions than among those with ≤50% attendance (Table 4).

**Table 4:** Attendance *tertiles* and test mean scores

	Attendance ≤ 50%	Attendance > 50%	*p*-value
Mean score in performance (n)	56.4 ± 19.8 (719)	66.7 ± 19.6 (685)	< 0.0001
Lower *tertile*^a^ percentage (n)	14.6 (105)	6.6 (45)	< 0.0001
Mean lower *tertile* score	25.6 ± 8.2	25.2 ± 6.2	0.759
Mid *tertile*^b^ percentage (n)	47.6 (342)	33.8 (232)	< 0.0001
Mean mid *tertile* score	49.7 ± 7.7	51.3 ± 7.6	< 0.012
Higher *tertile*^c^ percentage (n)	37.8 (272)	59.6 (408)	< 0.0001
Mean higher *tertile* score	76.7 ± 9.9	80.1 ± 10.1	< 0.0001

^a^students with ≤ 33.3% score in pharmacology

^b^students with > 33.3 to ≤ 66.6% score in pharmacology

^c^students with > 66.6% score in pharmacology

### Gender-based attendance and performance

The patterns of gender-based attendance and test score achieved are shown in Table 5. Compared to male students, female students showed significantly higher overall mean resource session attendance (55.1% ± 37.2% vs. 39.1% ± 35.1%). Female students had a lower percentage of zero attendance (20.2% vs. 29.9%) and had a higher percentage of 100% attendance (22.7% vs. 9.3%) compared to male students. These differences were statistically significant (*p* < 0.0001; Table 5). Although the female students had a greater tendency to attend structured educational activities, their total mean test scores did not significantly differ from those of male students.

**Table 5:** Patterns of gender-based attendance and test score achieved

Students’ Characteristics	Male	Female	*p*-value
Total mean attendance (n)	39.1 ± 35.1 (592)	55.1 ± 37.2 (812)	< 0.0001
Lower *tertile*^a^ percentage (n)	11.5 (68)	10.1 (82)	0.406
Lower *tertile* score	25.6 ± 8.0	25.3 ± 7.4	0.778
Mid *tertile*^b^ percentage (n)	43.1 (255)	39.9 (319)	0.154
Mid *tertile* score	50.6 ± 7.7	50.2 ± 7.8	0.480
Higher *tertile*^c^ percentage (n)	45.4 (269)	50.6 (411)	0.055
Higher *tertile* score	79.1 ± 10.5	78.6 ± 9.9	0.543
Total mean score (n)	60.7 ± 20.5 (592)	62.0 ± 20.2 (812)	0.219
0% attendance (n)	29.9 (177)	20.2 (164)	< 0.0001
Mean score	53.8 ± 18.0	55.5 ± 19.8	0.417
100% attendance (n)	9.3 (55)	22.7 (184)	< 0.0001
Mean score	72.6 ± 16.4	68.7 ± 17.0	0.130
Percentages of students with distinction^d^	8.4 (50)	8.0 (65)	0.766
Mean score	95.6 ± 4.0	95.2 ± 3.9	0.608

^a^students with ≤ 33.3% score in pharmacology

^b^students with > 33.3 to ≤ 66.6% score in pharmacology

^c^students with > 66.6% score in pharmacology

^d^students with grade ≥ 90%

## Discussion

It is well known that student attendance at classroom sessions in medical schools with PBL or lecture-based learning (LBL) curricula is on the decline globally [6–13, 26]. It is evident from our study that attendance at the optional resource session was very high at the start of the medical programme in unit I, but attendance declined as years progressed during the pre-clerkship phase (Fig. 1). This finding is consistent with that reported by Mattick et al. [26] among undergraduate medical students following LBL in the UK. Classroom absenteeism is influenced by student, teaching, and class/college environment-related factors [26–28]. Among the most frequent factors cited for absenteeism is a lack of interest in the topic discussed [27, 29], self-study preferences [30], inconvenient class schedules such as early morning lectures [14, 27, 30], dislike of teaching style [26, 27], online availability of lecture material [27], and classroom environment [27, 28].

In the context of CMMS-AGU, factors such as inconvenient class schedule, poorly ventilated/overcrowded lecture halls, and students’ low income can be excluded as reasons for absenteeism because resource sessions are held at noon (Table 2) in air-conditioned lecture halls with state-of-the-art audio-visual facilities. Approximately 95% of students had full scholarship support from their countries. A lack of intrinsic motivation may be possible; some students may not realize that the study of medicine is rigorous and challenging [14, 27]. Absenteeism, therefore, can be one of the convenient ways to evade the curriculum [31]. Ready access to PowerPoint files used as an instructional tool in resource sessions, along with the audio recording of lectures by some students, may be the main reason for absenteeism. In medical schools, the accessibility of online lecture contents has been reported to have a negative impact on students’ class attendance [11, 27, 32, 33]. A questionnaire survey of the students to study the actual reasons for absenteeism may provide better insight.

An growing body of evidence supports the positive correlation between classroom attendance and improved academic performance, such as conventional wisdom, across a wide variety of courses and colleges. This finding has been reported among pre-clerkship medical students in traditional curricula [6, 34], pharmacy students [35–37], students in obstetrics/gynaecology courses [10] and students in pharmacology courses in medical school [8, 38, 39]. Evidence-based data for this correlation in the PBL curriculum is lacking. The current study was conducted to determine the impact of resource session attendance on student achievement in the pharmacology knowledge component during the pre-clerkship learning environment that follows the PBL curriculum. A positive correlation was apparent between attendance and pharmacology achievement across all pre-clerkship phase units. The more resource sessions the student attends per unit, the higher the score achieved across all units (Table 3). This finding was further supported by the following: a) a lower *tertile* percentage that was significantly lower among students who had attended > 50% of the resource sessions and b) a higher *tertile* score that was substantially higher in students who attended > 50% of the resource sessions (Table 4). Therefore, resource session attendance appears to be one of the many critical determinants of the achievement of pharmacology learning outcomes by pre-clerkship medical students in the PBL curriculum. Our findings are in line with those of several studies conducted to assess pharmacology performance in medical schools with LBL curricula [8, 38, 39]. Among second-year medical students, high lecture attendance was found to be associated with higher examination scores [38]. A significant positive correlation was found between attendance and academic performance in pharmacology theory and practical examinations in second-year medical students [39]. Hamdi [8] reported that absenteeism had a significant effect on medical pharmacology achievement by fourth-year students, and the author emphasized the importance of regular attendance as an effective way of improving test scores.

The gender-related correlation between attendance and academic achievement in medical school is unclear [21]. The current study revealed that female students had significantly higher total mean (and 100%) attendance than male students. Nonetheless, no significant gender difference was evident concerning the overall mean score achieved and the distinction grade ≥ 90% in pharmacology (Table 5). Table 6 shows an association between the students’ performance in pharmacology and the percentage of attendance (*p*-value < 0.001), while there is no association between students’ performance in pharmacology and gender. Female students had to attend classes more frequently to earn scores comparable to those of males. These findings are consistent with those of published studies [35, 40]. Daud et al. [40] studied the impact of class attendance on test performance in community medicine of fourth-year medical students in LBL curricula and showed that male students had a significantly lower percentage of class attendance than female students; furthermore, a nonsignificant gender difference in scores was found. Cortright et al. [21] studied the effect of class attendance on gender differences in physiology performance and reported that the grades achieved above and below the class average by female but not male students were directly related to the number of classes attended.

**Table 6:** Association between Students’ Performance in Pharmacology and percentage attendance and gender

		Lower *Tertiles*		Mid *Tertiles*		Higher *Tertiles*		Total	Chi-Square test (Value, *P*-value)
		no.	%	no.	%	no.	%		
Attendance %	0	51	15.0	182	53.4	108	31.7	341	(108.71, < 0.001)
	1–24	22	19.5	53	46.9	38	33.6	113	
	25–49	27	13.4	84	41.6	91	45.0	202	
	50–74	23	9.1	102	40.3	128	50.6	253	
	75–99	21	8.2	72	28.1	163	63.7	256	
	100	6	2.5	81	33.9	152	63.6	239	
Gender	Female	82	10.1	319	39.3	411	50.6	812	(3.71, 0.156)
	Male	68	11.5	255	43.1	269	45.4	592	
	Total	150	10.7	574	40.9	680	48.4	1404	

Nevertheless, our findings differ from those reported by others [7, 41]. Bamuhair et al. [41] reported that no significant differences were observed concerning cardiology examination performance between male and female medical students in a PBL curriculum, although male students had slightly higher but statistically nonsignificant percentages of lecture attendance. In another study, continuous score assessment for second-year pharmacology students revealed that females achieved significantly higher total mean scores, although there was no significant gender difference in lecture attendance [7]. Based on the current study design, the plausible explanation for such gender differences is uncertain, but it may be attributed to various parameters, such as motivation, learning style preference, and self-regulated learning behaviour. These variables merit further research from the PBL perspective. Of note, students who attended classes are often those who are intrinsically motivated and have a genuine desire to learn [2, 21]. It is plausible that females may be more motivated, as suggested by their attendance, than male students (Table 5).

In contrast, male students may perceive that attending resource sessions has minimal impact on their grades. A focus group study may help to resolve this issue. Textbooks and other online supplements, audio-recorded lectures and faculty PowerPoint files of each resource session in pharmacology may offer useful alternative tools to support self-regulated learning behaviour and, hence, to attain grades comparable to those earned by female students.

The strength of this study is that the sample size of the pre-clerkship phase students (1404 students) is robust and included all pre-clerkship phase students of a PBL medical curriculum. The limitation of this study is that student attendance was monitored using paper-based attendance registers signed by the students. This approach has disadvantages because the time taken for data collection reduces the lecture time and may lead to fake attendance by some students. The biometric method for recording classroom attendance is preferred. This study also did not evaluate student performance in other domains, including skills and their ability to integrate pharmacology concepts with other basic and clinical disciplines. A mixed-methods approach using both quantitative and qualitative methods would have been helpful to delineate the role of factors other than classroom attendance in explaining test performance. There is a considerable lag between the data collection and publication; thus, the findings may not necessarily reflect the current situation in the institution in which the study was performed. Moreover, the associations found in the study may not be robust because a multivariate analysis was not used to exclude confounding factors that could affect absenteeism and performance.

## Conclusions

The present study highlights a significant positive correlation between resource session attendance and test scores achieved in pharmacology by pre-clerkship medical students in a PBL curriculum. Although female students showed a greater commitment to attend resource sessions, the overall gender-based score achieved was not statistically significant. Female students had to attend the resource session more frequently to earn comparable scores to those achieved by male students. The study did not permit a rational explanation for these findings. Further studies using mixed methodology are required to explore the gender-based variation concerning students’ intrinsic versus extrinsic motivation, learning style preferences and self-regulated learning behaviours to better understand the learning process of medical students in the PBL curriculum. A questionnaire survey of the students may be required to study reasons for their absenteeism.

## Data Availability

The data sets used and analyzed during the current study are available from the corresponding author on request.

## Abbreviations

AGU: Arabian Gulf University
CMMS: College of Medicine & Medical Sciences
LBL: lecture-based learning
MCQs: multiple-choice questions
OSPE: Objective Structured Practical Examination
PBL: Problem-based learning
r: correlation coefficient
SAQs: short answer questions
